# Linking differences in action perception with differences in action execution

**DOI:** 10.1093/scan/nsu161

**Published:** 2015-03-13

**Authors:** A. Macerollo, S. Bose, L. Ricciardi, M. J. Edwards, J.M. Kilner

**Affiliations:** ^1^Sobell Department of Motor Neuroscience and Movement Disorders, UCL Institute of Neurology, London, WC1N 3BG, UK,; ^2^Department of Neuroscience and Sense Organs, University of Bari, Bari, Italy, and; ^3^Department of Clinical and Experimental Medicine, University of Messina, Messina, Italy

**Keywords:** mirror neurons, action observation, action perception, perception deficits, movement disorders

## Abstract

Successful human social interactions depend upon the transmission of verbal and non-verbal signals from one individual to another. Non-verbal social communication is realized through our ability to read and understand information present in other people’s actions. It has been proposed that employing the same motor programs, we use to execute an action when observing the same action underlies this action understanding. The main prediction of this framework is that action perception should be strongly correlated with parameters of action execution. Here, we demonstrate that subjects’ sensitivity to observed movement speeds is dependent upon how quickly they themselves executed the observed action. This result is consistent with the motor theory of social cognition and suggests that failures in non-verbal social interactions between individuals may in part result from differences in how those individuals move.

## INTRODUCTION

There is now substantial evidence that cortical regions known to be important in encoding parameters of executed actions are also active when observing another person performing the same action. It has been proposed that this activity is important for non-verbal social communication (James, 1890; Jeannerod, 1994; Prinz, 1997; Blaffer-Hrdy, 2009; Frith and Frith, 2012). The most well known example of this is mirror neurons (Kilner and Lemon, 2013). Mirror neurons were first described in the rostral division of the ventral premotor cortex (area F5; di Pellegrino *et al.*, 1992; Gallese *et al.*, 1996) and have subsequently been reported in the inferior parietal lobule (Fogassi *et al.*, 2005) dorsal premotor cortex (Dushanova and Donoghue, 2010) and primary motor cortex (Vigneswaran *et al.*, 2013). Mirror neurons are a class of neuron that modulate their activity both when an individual executes a specific motor act and when they observe the same or similar act performed by another individual. These findings in macaque monkeys are supported by a large number of human neuroimaging studies that provide evidence for a remarkable overlap between brain regions recruited during action execution and action observation (Grèzes and Decety, 2001; Rizzolatti and Craighero, 2004; Caspers *et al.*, 2010; Molenberghs *et al.*, 2012 for reviews).

Ever since their discovery, it has been proposed that mirror-neurons, and motor system activity during action observation more generally, underlie our ability to ‘understand’ actions (Gallese *et al.*, 1996; Fogassi *et al.*, 2005). However, despite decades of research there is still much debate about this proposed role of the motor-system in action perception and inference (Hickok, 2009; Gallese *et al.*, 2011). This is of particular interest as all major theoretical accounts of the role of the motor system in action perception and inference predict a tight link between neural activity in the motor-system during action observation and our ability to execute the same action (Wolpert *et al.*, 2003; Rizzolatti and Craighero, 2004; Kilner *et al.*, 2007; Friston *et al.*, 2011). The key concept in all these theoretical models is that the same motor models employed during action execution serve as the basis for perception and inference during action observation. Previous research has provided some evidence that is consistent with this hypothesis. For example, it has been demonstrated that gross deficits in action production resulting from either cortical lesions and/or apraxia are correlated with deficits in both action recognition and imitation (Buxbaum *et al.*, 2005; Negri *et al.*, 2007; Tessari *et al.*, 2007; Pazzaglia *et al.*, 2008). All these studies correlated differences in action perception with gross errors in action production, as scored by an expert observer. However, the proposed theoretical models make a much more specific prediction. According to the theoretical framework, the more similar two people execute the same action the more effective their ability to read and predict each other’s actions will be. In other words, differences in action inference between individuals should be correlated with subtle differences in the kinematics of how individuals execute the action they are observing. Here, we tested this prediction. The aim of this study was to test whether between subject differences in the parameters of the kinematics of an executed action correlated with differences in action perception when observing the same action. To this end, we deliberately tried to maximize between subject variance in the kinematics of a simple reach and grasp action by testing a heterogeneous population of individuals that included healthy subjects of different ages, as well as different movement disorder patients. We demonstrate that subject’s sensitivity to observed actions is indeed dependent upon how they themselves executed the observed action.

## METHODS

Seventy-two subjects were recruited for the study, 16 young healthy adults, 25 movement disorder patients, 15 functional movement disorder (FMD) patients and 16 age matched controls. Three movement disorder patients had difficulties performing the task successfully and were excluded from further analyses, leaving a total of 69 subjects with 22 movement disorder patients (Tables 1–4). All subjects gave signed consent and the study was approved by a local ethical committee.

**Table 1:** Individual profiles for young healthy subjects tested in the study

Subject number	Gender	Age	Left/right handed
1	Female	24	Right
2	Female	33	Right
3	Male	33	Right
4	Male	30	Right
5	Male	35	Right
6	Male	30	Right
7	Female	24	Right
8	Female	32	Right
9	Female	30	Right
10	Male	21	Right
11	Female	30	Right
12	Male	37	Right
13	Male	34	Right
14	Female	27	Right
15	Male	32	Right
16	Female	30	Right

**Table 2:** Individual profiles for age-matched healthy subjects tested in the study

Subject number	Gender	Age	Left/right handed
1	Female	73	Right
2	Female	68	Right
3	Male	70	Right
4	Female	64	Right
5	Female	55	Right
6	Female	63	Right
7	Female	38	Right
8	Male	73	Right
9	Female	65	Right
10	Male	55	Right
11	Female	48	Right
12	Female	48	Right
13	Male	53	Right
14	Female	69	Right
15	Female	68	Right
16	Female	71	Right

**Table 3:** Clinical profiles for patients tested in the study

Subject number	Gender	Age	Left/right handed	Diagnosis
**1**	Male	71	Right	IFD
**2**	Female	61	Right	ISD
**3**	Female	66	Right	PD
**4**	Female	77	Right	ISD
**5**	Female	41	Left	ISD
**6**	Male	59	Right	PD
**7**	Male	60	Right	DT
**8**	Female	65	Right	DT
**9**	Female	74	Right	ISD
**10**	Female	69	Right	PD
**11**	Female	69	Right	ISD
**12**	Female	61	Right	PD
**13**	Female	66	Right	IFD
**14**	Female	33	Right	ISD
**15**	Male	70	Right	PD
**16**	Female	69	Right	ISD
**16**	Female	45	Right	PD
**18**	Female	42	Right	ISD
**19**	Male	70	Left	PD
**20**	Male	58	Right	PD
**21**	Female	50	Right	ISD
**22**	Female	53	Left	IFD

PD, Parkinson’s disease; DT, Dystonic tremor; IFD, Idiopathic focal dystonia; ISD, Idiopathic segmental dystonia.

**Table 4:** Clinical profiles for patients with FMDs in the upper limbs tested in the study

Subject number	Gender	Age	Left/right handed	Diagnosis
1	Male	61	Right	FT
2	Male	48	Right	FD
3	Female	53	Right	FT
4	Male	53	Right	FT
5	Female	30	Right	FD
6	Female	43	Right	FT
7	Male	45	Right	FD
8	Female	66	Right	FT
9	Female	53	Right	FT
10	Male	39	Right	FT
11	Female	19	Right	FD
12	Female	58	Right	FT
13	Female	46	Right	FT
14	Male	58	Right	FD
15	Male	63	Left	FT

FT, Functional tremor; FD, Functional dystonia.

The patients were diagnosed with Parkinson’s disease (PD), FMDs (phenotypes tremor or dystonia) or organic movement disorders (dystonia and dystonic tremor) as follows.

The patients classified as affected by PD fulfilled the United Kingdom Queen Square Brain Bank diagnostic criteria (Hughes, *et al.*, 1992).

Primary dystonia is a condition characterized by abnormal postures in the limb. These patients were classified as affected by focal or segmental dystonia following the new guidelines by Albanese *et al.* (2013).

The FMDs involved in this study had either a functional tremor (FT) or functional dystonia (FD). Patient with FT present most frequently with tremors of the hands and arms, but tremor of the head, legs and palate can also occur. Peculiarly, it is present at rest, posture and on action. It is characterized by constant variability in amplitude and frequency, clear distractibility with clinical maneuvers and entrainment with movement of other limb (especially ballistic movements). Furthermore, it paradoxically worsens with loading.

FD is the second most common presentation in patients with FMDs. The typical presentation is with fixed abnormal postures at rest, rather than the typical mobile postures of organic dystonia, showing an unusual distribution of dystonia given the age at onset. The usual precipitating factor is a minor peripheral trauma, and term of ‘causalgia-dystonia’ was coined to classify this group (Bhatia *et al.*, 1993). Important features of FD are the absence of task or position specificity, the absence of sensory geste and poor response to botulinum toxin. The patients were classified as affected by FMDs following the formal diagnostic criteria by Fahn and Williams (1988), recently revised by Gupta and Lang (2009).

### (a) The observation task

The task is identical to one previously employed (Patel *et al.*, 2012). In brief, subjects were asked to watch a series of video clips showing the hand movements of two anonymized actors (one male and one female). The videos showed the actors performing a visual discrimination task. Here, we provide details of the task that the actors performed. It is important to note that the subjects in this study did not have any knowledge of the task that the actors performed. In this task, the actors were shown two images in swift succession on a computer screen. Each image consisted of six Gabor gratings (circular patches of smoothly varying light and dark bars) arranged around a central fixation point. The background was a uniform grey screen of luminance 3.66 cd m^−2^ (see electronic Supplementary Material, Figure S1a). In one of the two images, all of the Gabor gratings were set at the same contrast (‘baseline Gabors’), but in the other image, one of the Gabors was set to be a higher contrast than the other five ‘baseline’ Gabors, and appeared as a ‘pop-out’. ‘Baseline’ Gabors were displayed at a contrast of 20% (where 0% was not a visible difference between the light and dark grating bars and 100% is the maximum difference). The ‘pop-out’ Gabors varied in contrast between 23 and 80%, in increments of 3%. The appearance of the ‘pop-out’ Gabor in either the first or second image, its contrast and its spatial position (orientation around the central point) in each trial varied randomly throughout the experiment.

After presentation of the two images, the actor was required to make a decision as to which image (first or second) they believed contained the ‘pop-out’. After each decision, actors were asked to rate their confidence on a scale of 1-6 (1 denoting lowest possible confidence). The actors were required to express their choice by using their dominant hand on a custom-made response board (Supplementary Figure S1b). The board comprised four separate sensors: a sensor on which the hand rested between each trial, a sensor on which a marble was placed, and two sensors, each within holes equidistant from the resting position of the marble that sensed when the marble was placed into the hole. After the presentation of the two images a grey screen appeared with the numbers ‘1’ and ‘2’. To convey their forced decision as to which image contained the ‘pop out’ Gabor, the actor was required to move the marble from its rest position in the centre of the board and place it on either the left or right hole, corresponding to the first or second image, respectively. Actors were given no instruction as to how fast or slow to move the marble. Depending on where the marble was placed (after the marble and hand are returned to their original rest positions on the board), a red square frame appeared around either ‘1’ or ‘2’ on the computer screen to highlight the participant’s decision. Following this, an additional grey screen with the numbers ‘1’-‘6’ appeared, requiring the participant to rate their confidence in the decision they have just made on a scale of 1-6 (1 being least confident). This rating was expressed using the numerical keys of the QWERTY keypad on the laptop using their non-dominant hand (i.e. the hand that is not placed on the sensor), and subsequently a red square frame appeared around the selected rating.

The contrast of the ‘pop-out’ Gabor was adjusted throughout the experiment using a two-up, one-down staircase procedure such that all participants converged onto a final score of ∼71% correct (correct referring to making the right decision as to which of the two images contained the pop-out). The staircase operated such that after two consecutive correct decisions the contrast was decreased by one step, whereas after one incorrect decision the contrast was increased by one step. This was done to ensure that the analysis of movement time (MT) and confidence was not affected by performance. It also helped one to ensure that actors used the full extent of the confidence scale.

All movements of the actors in every trial were videoed, and these videos were edited for presentation to the subjects in this experiment. All video clips were edited to start 300 ms prior to onset of the movement and ended once the decision had been made. In this way, we produced 390 unique videos that showed only the right hand of the actor reaching and grasping a marble and moving it to one of the two decision holes from a third person perspective. In addition, this dataset also included the 390 confidence ratings of the actors in their decision.

In this study, all subjects observed the 390 videos in a randomized order. After watching each video clip, subjects were asked to rate how confident they felt the actor in the video was in their decision. They did this using the numerical keys 1-6 of the QWERTY keypad on the laptop using their non-dominant hand, where 1 was least confident. No feedback was given for this task. The experiment consisted of 390 trials that were presented in a random order. Subjects performed three blocks of 100 trials and one block of 90 trials, with an interval between each.

### (b) Execution condition

To measure, how each subject executed the action, we asked each subject to make 10 movements to reach and grasp the marble and move it to the left hole and 10 movements to the right hole. No instruction was given as to the speed subjects should move. Here, for each trial we calculated the time from movement onset to the time that the marble was placed in the left and right hole. As the distance was travelled was constant, we used MT as an estimate of movement speed.

### (c) The visual control condition

In the visual control condition subjects observed a series of videos that showed a dot moving from left to right across the screen. At some fixed point the dot disappeared behind an occluder. The subjects’ task was to the press the space bar when they thought the dot would reappear. The dot never reappeared and no feedback was given as to the accuracy of the response. Twenty-five different movement speeds were tested that overlapped with the movement speeds of the observed actions. Subjects completed two blocks of 25 trials. All trials where the estimated time of reappearance differed from the actual time by greater or ∼1000 ms were treated as outliers and were not included in further analyses. The aim of the visual control condition was to test whether subjects could perceive different movement speeds. To test this, we correlated the estimated time of reappearance with the actual time of reappearance. If subjects could perceive different movement speeds then the estimated and actual reappearance times would be linearly correlated.

## ANALYSIS

To test for modulations in inferred confidence with movement speed the 390 observed videos were sorted by movement speed from the fastest to the slowest. The mean inferred confidence was then calculated for consecutive 39 sorted videos producing 10 mean movement speeds and mean inferred confidence levels for each subject. To test for sensitivity to the observed movement speed, we calculated the change in confidence with observed movement speed by calculating the slope between these two measures. This was performed initially across all observed videos and subsequently over only the fastest half of the videos.

## RESULTS

**Fig. 1:**
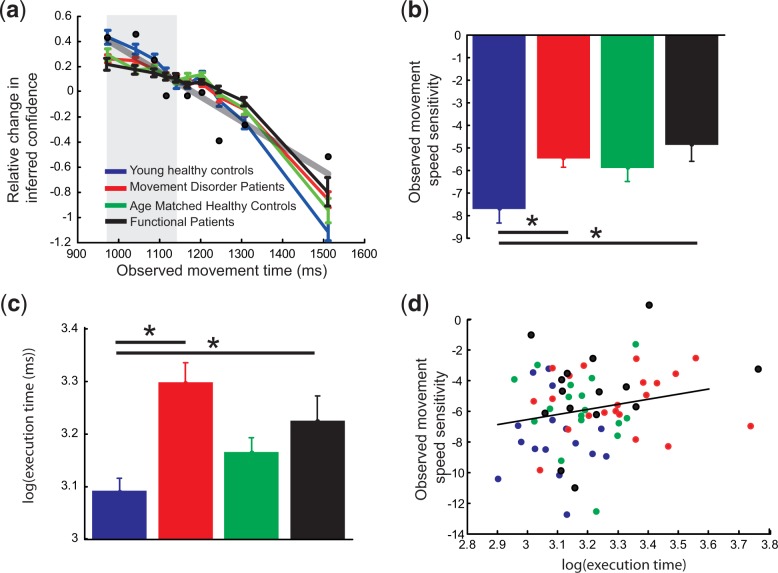
Through the whole figure blue shows data from the young healthy controls, red the movement disorder patients and green the age matched healthy controls. (**a**) shows the relationship between observed movement speed and relative change in inferred confidence. The black dots show the actual confidence of the person being observed and the grey line shows the linear fit of this data. Note that the data for each subject was mean corrected prior to averaging. (**b**) show the average sensitivity to the observed movement speed for the different groups. (**c**) shows the average movement speed of the subjects during action execution. (**d**) show the correlation of the execution time against observed movement sensitivity for all subjects. The black line shows that the results of a linear regression between these two variables. All error bars are standard errors of the mean. *, indicate significant differences at *P* < 0.05.

Previous research has demonstrated that subjects are able to correctly infer the subjective confidence of another person simply from the kinematics of their observed action, with faster actions being perceived as more confident (Patel *et al.*, 2012). Here, we tested with 69 subjects whether people’s sensitivity to the observed movement speed is dependent upon how they themselves executed the observed action. To increase variance in execution speed across the population, we tested four different groups of subjects (Tables 1–4), 16 young healthy subjects [mean age 30.1 (+/− 4.4) years], 22 movement disorder patients [mean age 60.4 (+/− 11.8) years], 15 FMD patients [mean age 49.4 +/− 12.4] and 16 age matched healthy controls [61.1 (+/− 10.6) years]. All 69 subjects modulated their estimate of the actors’ confidence level by the speed of the observed action, with faster actions being rated as more confident [Figure 1a; main effect of time F(9585) = 251.1, *P* < 0.05]. All subjects showed a negative relationship between observed movement speed and inferred confidence. This relationship was significant in 67/69 subjects (*P* < 0.05). Importantly, the nature of the relationship between observed MT and inferred confidence was significantly modulated by subject group [Interaction between observed MT and group F(27 585) = 2.59, *P* < 0.05]. There was no significant main effect of subject group [F(3,65) = 0.78, *P* = 0.5].

To investigate, this effect further, we calculated the sensitivity of each subject to the observed movement speed of the actor. As inferred confidence decreased with decreasing observed movement speed (increasing observed MT), we estimated subjects sensitivity to the observed movement speed by calculating the slope between the observed movement speed and inferred confidence. The logic being that subjects that could not observe any difference in the observed movement speeds should have no difference in their level of inferred confidence. Over all observed movements the movement disorder patients and the FMD patients showed a lower sensitivity (flatter gradient) to the observed movements speeds than young healthy controls [Figure 1b; *t*(36) = −3.11, *P* < 0.05 and *t*(29) = −2.87, *P* < 0.05, respectively]. There was no significant difference in sensitivity between the movement disorder patients and the healthy age-matched controls [*t*(36) = −0.59, *P* = 0.56], and there was a trend to significance between young healthy controls and age-matched controls [*t*(30) = −2.03, *P* = 0.051].

The aim of this study was to test if any difference in sensitivity between subjects could be accounted for by differences in how the subjects executed the observed action. To test this end, we asked all subjects to perform the reach and grasp action and calculated their average movement speed. As expected, young healthy subjects executed the actions faster than both the movement disorder patients and the FMD patients [Figure 1c, *t*(36) = −4.15, *P* < 0.05 and *t*(29) = −2.52, *P* < 0.05, respectively]. In addition, there was a trend to significance between the movement disorder patients and the age-matched controls [*t*(36) = −1.93, *P* = 0.06] and between young healthy controls and age-matched controls [*t*(30) = −2.01, *P* = 0.053]. No other contrasts showed evidence of a significant difference or trend to significance.

**Fig. 2:**
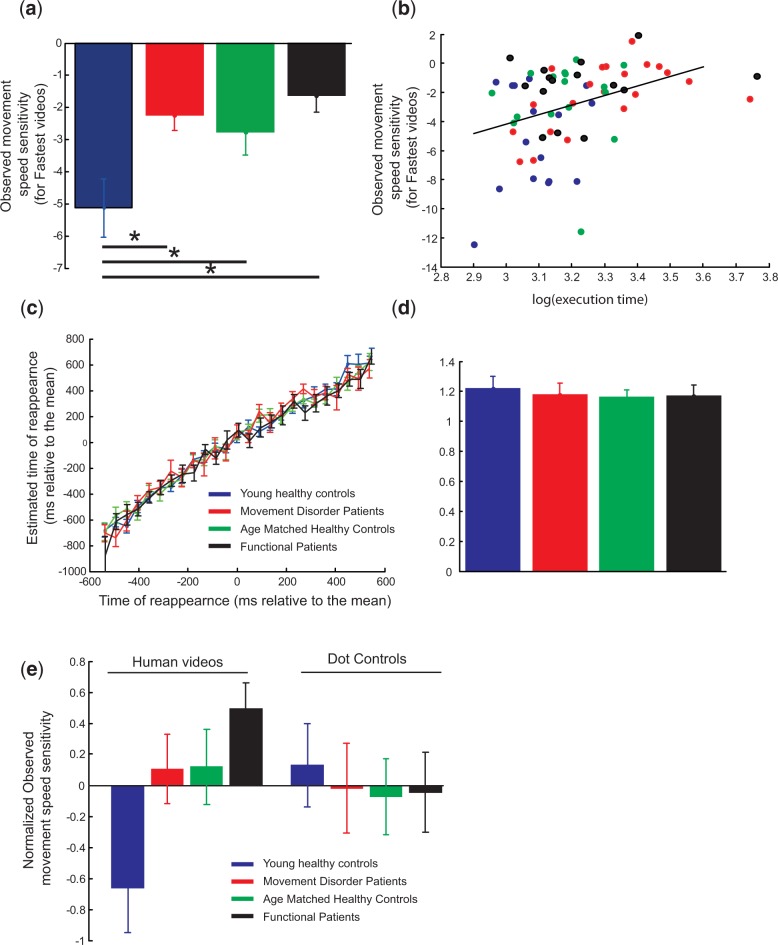
Through the whole figure blue shows data from the young healthy controls, red the movement disorder patients and green the age matched healthy controls. (**a**) show the average sensitivity to the observed movement speed for the different groups. (**b**) shows the correlation of the execution time against observed movement sensitivity for all subjects. The black line shows that the results of a linear regression between these two variables (**c**) show the correlation of estimated time of dot reappearance and actual time of dot reappearance for all three groups. (**d**) shows the average slope of the regression between actual time of reappearance and estimated time of reappearance for each subject in the different groups. (**e**) shows the average sensitivity to the observed movement speed and the observed movement speed for the different groups. Note that this data has been normalized prior o plotting such that the mean was equal to one and the standard deviation equal to one. All error bars are standard errors of the mean. *, indicate significant differences at *P* < 0.05.

To test any relationship between executed movement speed and sensitivity to observed movement speed, we correlated the two measures. There was no significant relationship found between the two measures (R^2 ^= 0.045, *P* = 0.07; Figure 1d). However, this initial analysis assumed that the sensitivity of the observer to the observed MTs would be the same across all observed MTs. This is in distinction to the predictions of the theoretical work where the observer should be least sensitive when movement speeds were most different to the speed the observer would execute the action. To test this, we calculated the sensitivity of the observer to the fastest half of the observed movement speeds (grey box Figure 1a). For the fastest observed movements there was a significant difference in mean sensitivity to the observed MTs between healthy young adults and movement disorder patients [Figure 2a; *t*(36) = −3.1, *P* < 0.05], between FMD patients and healthy young controls [*t*(29) = −3.4, *P* < 0.05] and between healthy young controls and age-matched controls [*t*(30) = −2.07, *P* < 0.05]. There were no significant differences between any of the other contrasts. To test for the relationship between sensitivity to observed MTs and executed MTs, we correlated the two measures. When inferring on the fastest 50% of the observed movements there was a significant correlation between sensitivity and executed movement speed [Figure 2b, parametric R^2 ^= 0.14, *P* < 0.05; non-parametric R^2 ^= 0.15, *P* < 0.05]. Thus, as predicted sensitivity to observed movement speed was correlated with execution speed across subjects.

To rule out this result being explained by modulations with other correlated factors, subjects age and overall estimated mean confidence level, we performed a stepwise regression to explain sensitivity to observed movement speed with execution speed, subjects age and subject mean estimated confidence in the model. Of these three factors subjects’ execution speed explained the most variance in the sensitivity to the observed movement speed and the final model from this stepwise regression included the subjects’ movement speed and the mean estimated confidence level in the final model (R^2 ^= 0.28, *P* < 0.05). The final model excluded subjects’ age as not explaining sufficient extra variance to be included.

Here, we have demonstrated a significant relationship between subjects’ sensitivity to observed movement speed and their own movement speed during action execution. To test whether this was a general lack of sensitivity to movement speed or was more specific to observing human movements we asked subjects to perform a control condition. Here, subjects observed a dot moving with constant velocity from left to right across the screen. After a certain time the dot disappeared behind a screen. The subjects’ task was to press a button when they thought the dot would reappear from behind the screen. The range of dot speeds was chosen to overlap the observed mean movement speeds. To estimate whether subjects correctly perceived the dot speed, we correlated the estimated time of reappearance with the actual time the dot would have reappeared. If subjects could correctly perceive and estimate the dot speed these two measures should be linearly related across different dot velocities and the gradient should be close to unity. There were no significant differences in the gradients between these two measures across the different subject groups. [Young adults *vs* movement patients *t*(27) = 0.36, *P* = 0.72, young healthy adults *vs* age matched controls *t*(30) = 0.56, *P* = 0.58; movement disorder patients *vs* age matched controls *t*(27) = − 0.15, *P* = 0.88; FMD patients *vs* young healthy adults *t*(29) = 0.46, *P* = 0.65; FMD patients *vs* age matched controls *t*(29) = −0.07, *P* = 0.94; FMD patients *vs* movement disorder patients *t*(26) = 0.07, *P* = 0.94; Figure 2c and d].

To show the dissociation between inferring on movement speeds in the control task and the movement speeds when observing actions, we tested whether there was an interaction between subjects’ sensitivity to observed action and to the dot movements and the subject group. To this end, we first normalized the data for the two tasks as the two tasks had different measures of sensitivity. For each task the data were normalized such that that mean was equal to zero and the standard deviation equal to one. Importantly, there was a significant interaction between the measure of sensitivity for observed human movements and dot movements and subjects group [F(3,56) = 3.18, *P* = 0.031, Figure 2e]. The interaction was driven by a more negative gradient (increased sensitivity; Figure 1a and b) for the young healthy controls when inferring on the videos of another human compared with other groups. There were no significant main effects [main effect of group F(3,56) = 1.15, *P* = 0.34; main effect of task F(1,56) = 0.007, *P* = 0.93].

## DISCUSSION

There is a lack of agreement as to the functional role, if any, of activity in the motor-system during action-observation (Hickok, 2009; Gallese *et al.*, 2011). The discovery of mirror neurons in ventral premotor cortex and the extensive overlap in regions of the motor system active during both action observation and action execution in humans has led many to assert that we require our motor systems to ‘understand’ observed actions (di Pellegrino *et al.*, 1992; Gallese *et al.*, 1996; Gallese *et al.*, 2011). The main prediction of this action ‘understanding’ functional framework is that the sensitivity of a subject to correctly perceive information present in an observed action should be strongly correlated with how the subject executes the same action. Here, we have shown precisely this. Moreover, we have demonstrated that this difference in inference is not due to a general deficit in perceiving movement speed but was specific to observing human biological actions.

Here, we have tested both healthy control subjects, patients with organic movement disorders, including PD, dystonia and tremor patients, and patients with FMDs. The reason for using movement disorder patients was to increase the variance in the movement execution times across the population of subjects. Indeed, we found no evidence that the different patient groups performed any differently and indeed many of the patients showed no difference in their execution times from age-matched healthy controls (Figure 2b). Even those patients that did perform slower were not severely impaired in this task. It is important to stress that here the aim was not to have carefully controlled patient groups but rather we deliberately choose a heterogeneous population of subjects so as to increase movement speed variance between subjects. For example, here we did not control for, or assess, the cognitive capabilities of the subjects and we did not control for time from illness or the therapy the patients had undergone. These factors would typically be controlled in any patient study to ensure that any between group differences could not be confounded by of any of these less interesting factors. There are a number of reasons why these patient measures could not explain our data. Here, we show a correlation across all subjects, irrespective of their health or disorder. Therefore, by maximizing the variance in the subject population we also minimize the chance that any result is due to a confound that occurs in just one of the patient groups. For example, there were only eight PD patients out of 69 subjects. Therefore, it is unlikely that any differences in medication or cognitive decline in this group would confound the correlation result across all 69 subjects. The other critical part of the study is that there was a significant interaction in the subjects’ ability to infer simple movement speed and perceive confidence from an observed action. In other words, all subjects could accurately perceive motion and velocity, but they differed in how they inferred information present in an observed action in a manner that was correlated with their own movement speed.

Previous studies of action-observation have focused on the functional role of the motor-system, specifically mirror neurons, in inferring the goal or intention of the action (di Pellegrino *et al.*, 1992; Gallese *et al.*, 1996; Umilta *et al.*, 2001). This is in distinction to the study described here in which we demonstrate a dependency between the kinematics of an executed action and subjects’ ability to ‘read’ information present in the kinematics of an observed action. However, these differences in the level of inference are not incompatible. Actions can be described at multiple levels and therefore there are multiple levels at which information about an observed action can be inferred. For example, actions can be described at least four levels: (i) the kinematic level: the trajectory and the velocity profile of the action, including both the reach and grasp phase of a goal directed action; (ii) the motor level: the processing and pattern of muscle activity required to produce the kinematics; (iii) the goal level: the immediate purpose of the action, for example to grasp an object and (iv) the intention level: the overall reason for executing the action. These levels are clearly non-independent and can be organized hierarchically, with the kinematics being dependent on the motor level, the motor level being dependent on the goal level and the goal level being dependent on the intention level. As one moves up the hierarchy, the action is described in more and more abstract terms. Although, this hierarchical organization enables inference at different levels any inferences must be based on the exteroceptive information of how the hand and arm move through space, as this is the only information the observer receives during action observation. In other words, whatever level an inference is made on an observed action, be it an inference on the goal or intention of the action or be it an inference on the emotional state of the observed person, whether they are anxious or confident, it is dependent upon the kinematics of the observed action. Indeed, recent theoretical predictive coding accounts of motor system activity during action observation (Kilner *et al.*, 2007; Friston *et al.*, 2011; Kilner, 2011), propose that the role of the motor system during action observation is to generate a prediction of the kinematics of the observed action. Indeed, previous research has highlighted the importance of kinematics in action perception both in healthy controls (Daprati *et al.*, 2007) and in patients (Castiello *et al.*, 2009). Although the results presented here are consistent with such theoretical account, it is important to note that we present no data that shows that the dependency between action execution and action inference demonstrated here is a result of activity in the motor system and this will form the basis of future studies.

## SUPPLEMENTARY DATA

Supplementary data are available at *SCAN* online.

## Conflict of Interest

None declared.

## Supplementary Material

Supplementary Data
