# Supplementary material for: Linking differences in action perception with differences in action execution
Source: Soc Cogn Affect Neurosci. 2015 Mar 13;10(8):1121–7. doi: 10.1093/scan/nsu161 (PMC4526482; doi:10.1093/scan/nsu161)
Supplement: Supplementary Data [file supp_10_8_1121__index.html]

Linking differences in action perception with differences in action execution — Linking differences in action perception with differences in action execution — Supplementary Data 

# Linking differences in action perception with differences in action execution

## Supplementary Data

files

**Files in this Data Supplement:**

- Supplementary Data - pdf file
- Supplementary Data - docx file
