# Supplementary figures and images for: Linking differences in action perception with differences in action execution
Source: Soc Cogn Affect Neurosci. 2015 Mar 13;10(8):1121–7. doi: 10.1093/scan/nsu161 (PMC4526482; doi:10.1093/scan/nsu161)

a

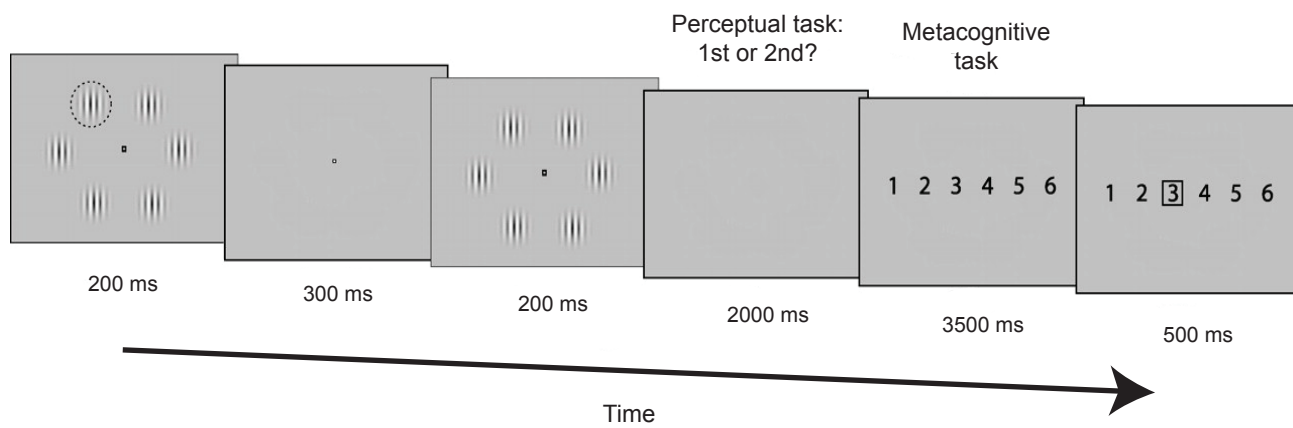

b

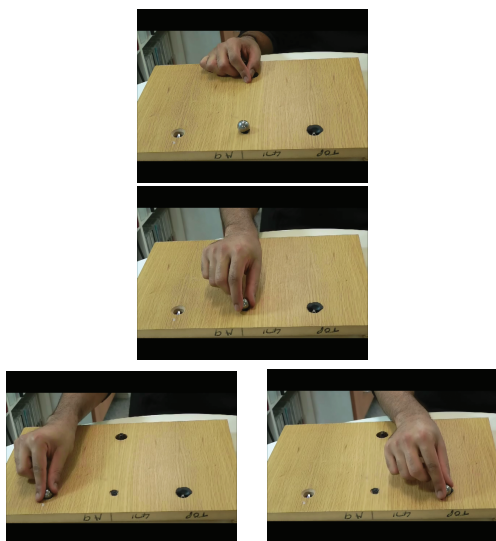

c

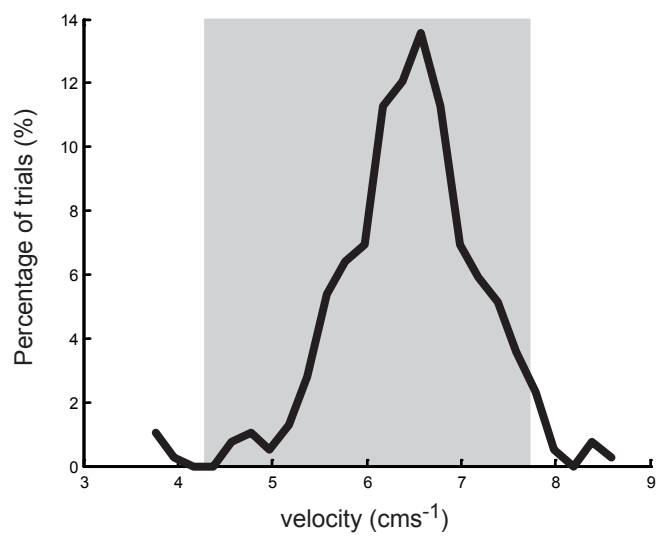

Supplement: Supplementary Data [file supp_nsu161_FigureS1.pdf]
