# Supplementary material for: Linking differences in action perception with differences in action execution
Source: Soc Cogn Affect Neurosci. 2015 Mar 13;10(8):1121–7. doi: 10.1093/scan/nsu161 (PMC4526482; doi:10.1093/scan/nsu161)
Supplement: Supplementary Data [file supp_nsu161_New_Microsoft_Office_Word_Document.docx]

**Supplementary Fig. 1.** **a)** shows the visual discrimination task that the actors performed. **b)** shows the response buttons. The stills are taken from two of the videos shown to the subjects. This same response apparatus was used in the execution condition. **c)** the overlap of movement speeds presented to the subjects for the observed movements of the actors (solid black line) and the speeds used for the visual control (grey box).
